# Development and validation of a core genome multilocus sequence typing scheme for *Citrobacter freundii*: application in outbreak investigations and comparative analysis across the *Citrobacter* genus

**DOI:** 10.1128/jcm.00860-25

**Published:** 2025-09-19

**Authors:** Bärbel Kieninger, Gabriel E. Wagner, Anca Rath, Anja Eichner, Jürgen Fritsch, Aila Caplunik-Pratsch, Jasmine Alikhani, Parham Heydarzadeh-Ghamsary, Adriana Cabal-Rosel, Werner Ruppitsch, Dag Harmsen, Mohammed R. Abdulla, Lena Ulm, Karsten Becker, Wulf Schneider-Brachert, Christian Kohler

**Affiliations:** 1 Department of Infection Prevention and Infectious Diseases, University Medical Center Regensburg, Regensburg, Germany; 2 Diagnostic and Research Institute of Hygiene, Microbiology and Environmental Medicine, Medical University of Graz 31475, Graz, Austria; 3 Austrian Agency for Health and Food Safety (AGES), Institute of Medical Microbiology and Hygiene 31190, Vienna, Austria; 4 Department of Periodontology and Operative Dentistry, University Hospital Münster 39069, Münster, Germany; 5 Friedrich Loeffler-Institute of Medical Microbiology, University Medicine Greifswald 26552, Greifswald, Germany; Maine Medical Center Department of Medicine, Portland, Maine, USA

**Keywords:** cgMLST, *Citrobacter freundii*, *Citrobacter portucalensis*, *Citrobacter braakii*, *Citrobacter europaeus*, outbreak, transmission, epidemiology

## Abstract

*Citrobacter freundii* is a nosocomial pathogen increasingly associated with multidrug resistance and hospital outbreaks. Despite its growing clinical relevance, no standardized core genome multilocus sequence typing (cgMLST) scheme has been available for high-resolution epidemiological analyses. Here, we developed and validated a robust cgMLST scheme for *C. freundii* comprising 3,250 target loci based on a curated data set of 825 globally distributed genomes representing extensive sequence type and geographic diversity. Validation against published outbreak data sets from hospitals in Finland and Belgium, as well as environmental and patient isolates from two German university hospitals, proved that the scheme possesses high target gene coverage (median 99.6%) and strong discriminatory power. Additionally, we developed a combined cgMLST scheme for *C. freundii*, C. *portucalensis*, C. *braakii*, and C. *europaeus*, based on 2,307 shared target loci and target gene coverages of ≥99.7%. This scheme proved suitable for cross-species outbreak analysis. Our analyses revealed environments, such as sinks, shower drains, and toilets, as likely reservoirs where *Citrobacter* species may persist in hospital settings. These findings suggest that environmental sources could play a significant role in transmission events involving patients, while allele-based cluster analyses indicated that direct patient-to-patient transmission was rare. Given the increasing prevalence of multidrug-resistant (MDR) *Citrobacter* strains, the level of discrimination achieved by these newly developed cgMLST schemes underscores the importance of accurate species identification and environmental screening in understanding the transmission dynamics of opportunistic healthcare-associated pathogens. Ultimately, this makes them valuable tools for genomic surveillance, outbreak investigation, and infection prevention.

**IMPORTANCE:**

Accurate identification and high-resolution typing of multidrug-resistant bacteria are essential for understanding their transmission dynamics in hospitals, particularly in light of the global spread of resistant strains and the role of environmental reservoirs. The newly developed cgMLST schemes presented here provide standardized, portable tools for both local and global scientific and clinical communities to conduct fine-scale genomic epidemiology across four *Citrobacter* species. These schemes support detailed outbreak reconstruction, source attribution, and cross-hospital comparisons, capabilities that are critical in an era of increasing antimicrobial resistance and international patient movement. By enabling consistent, species-specific surveillance and comparative analyses, cgMLST enhances infection control and public health responses, facilitating early detection and targeted intervention.

## INTRODUCTION

*Citrobacter freundii*, a member of the order Enterobacterales, has drawn increasing attention due to growing numbers of multidrug-resistant isolates, prompting its inclusion on the WHO Priority Pathogens List (1, 2). It is the most frequently reported species among *Citrobacter* infections and is primarily associated with urinary tract infections (61%), bloodstream infections (54%), and respiratory tract infections (29%) (3). In the hospital setting, their nosocomial transmission typically occurs via contact with contaminated surfaces (e.g., sinks, toilets), contaminated pharmaceuticals or medical devices, or the hands of healthcare workers (3).

A recent systematic review and meta-analysis reported a prevalence of up to 2.4% for *Citrobacter* species among nosocomial infections in hospitalized patients, with urinary tract infections, wound infections, and sepsis being the most common clinical presentations (3). Mortality rates ranged from 10% to 32%, particularly affecting immunocompromised individuals (3). In addition, the proportion of multidrug-resistant *Citrobacter* isolates has steadily increased in recent years, with multidrug resistance (MDR) rates exceeding 30% in some cohorts (3). This significantly limits therapeutic options. The ESCMID guideline recommends targeted therapy with novel β-lactam/β-lactamase inhibitor combinations, such as ceftazidime-avibactam or meropenem-vaborbactam, for infections caused by multidrug-resistant Gram-negative bacilli, including *Citrobacter* spp. (2).

Outbreaks involving *C. freundii* have been reported globally: Parida et al. described one in a neonatal unit in India (1979) (4); Pletz et al. traced an outbreak in Germany to contaminated vegetables (5); Hamerlinck et al. linked an outbreak in Belgium to the hospital water system (6); and Heljanko et al. identified *C. freundii* in both wastewater and patient samples in Finland (7). The pathogen also affects animals, including silver gulls in Australia (8).

To investigate such outbreaks, high-resolution typing methods are essential. Core genome multilocus sequence typing (cgMLST) based on whole genome sequencing (WGS) is one of the standard methods used for intra-species comparison. By analyzing allelic profiles of core genes, cgMLST enables standardized, high-resolution comparison of isolates, supporting outbreak detection and transmission analysis (9–12). However, no standardized cgMLST scheme for *C. freundii* has been published to date.

This study aimed to develop and validate such a scheme based on the following key criteria:

1. Core genome representation: Genomes from globally distributed isolates, including clinical and environmental sources, should be included. Existing schemes for *Enterococcus faecium* and *Mycobacterium tuberculosis* serve as positive examples where major population structures were successfully incorporated (13, 14).
2. Taxonomic consistency: Phylogenetic analyses often reveal discrepancies with traditional species definitions and frequent misclassifications, as seen in the development of a *Klebsiella oxytoca* scheme (15, 16).
3. Validation: Testing against published outbreak genomes is needed to confirm robustness and epidemiological utility (13, 17).
4. Allelic distance threshold: A threshold must be defined to infer transmission events and identify potential sources of infection.

## MATERIALS AND METHODS

### Sample collection and bacterial isolation

Samples originated from two university hospitals in Germany, University Hospital Regensburg (UHoR) and University Medicine Greifswald (UMG). At UMG, all *Citrobacter* spp. isolates collected from January to August 2024 underwent whole-genome sequencing (WGS; Data set 4). At UHoR, all *Citrobacter* spp. from blood cultures and MDR isolates from other sources have been collected since January 2020; all MDR isolates were sequenced, along with a randomly selected subset of blood culture isolates for surveillance purposes (Data set 5).

Samples were cultured on blood agar (UMG: BD, USA; UHoR: in-house) at 37°C for up to 24  h. Species identification was performed using MALDI-TOF MS (Bruker Biotyper), applying a score >2.0. Confirmed isolates were stored at −80°C.

### DNA extraction

Bacteria were cultured on blood agar (BD, USA) at 37°C for ≥16  h. Colonies were harvested with 1- µL inoculation loops (NerbePlus, Germany). DNA was extracted using the NucleoSpin Microbial DNA Kit (UMG; Macherey-Nagel, Germany) or the QIAamp DNA Mini Kit (UHoR; Qiagen, Germany). DNA quality and concentration were assessed using NanoDrop ND-1000 and Qubit 2.0. DNA was either processed for WGS or stored at −80°C.

### Whole-genome sequencing and assembly

At UMG, libraries were prepared with the Illumina DNA Prep Kit and sequenced on a MiSeq DX platform (2 × 300  bp, 2 × 250  bp, or 2 × 150  bp; Illumina, USA). A minimum coverage of 70× was applied. The consensus assembly was performed using the SeqSphere+ assembly pipeline. Briefly, quality control of RAW reads was performed with FastQC v0.11.7. Afterward, reads were adapter-trimmed and assembled using SKESA v2.3.0 (default mode) implemented in SeqSphere+ v10.0.0 (18, 19); and finally, the reads were remapped with BWA-MEM v0.7.15 to the assemblies and polished with the internal Ridom Typer algorithm.

At UHoR, library preparation used the Nextera DNA Flex Kit (Illumina, USA), and sequencing was performed on a NextSeq 550Dx (2 × 150  bp). Reads were processed using the AQUAMIS pipeline (20), which includes trimming (fastp), taxonomic classification (Kraken2), *de novo* assembly via Shovill (SPAdes v3.15.4 [21]), and quality assessment (QUAST, BUSCO). Resulting FASTA files were analyzed in SeqSphere+.

### Data sets and external sequences

All *Citrobacter* sequences used in this study originated from either the Genome Taxonomy Database (GTDB) database (August 2024, https://gtdb.ecogenomic.org/ [22]), European Nucleotide Archive (ENA, https://www.ebi.ac.uk/ena/), National Center for Biotechnology Information (NCBI, https://www.ncbi.nlm.nih.gov/datasets/genome/), or were newly assembled as part of this study.

To ensure consistent taxonomic classification, all sequences were reanalyzed using the GTDB-Tk tool (https://github.com/Ecogenomics/GTDBTk) (23). As a result, species designations throughout this manuscript follow GTDB nomenclature. Additionally, isolates from Data sets 4 (UMG) and 5 (UHoR) were analyzed using the NCBI AMRFinderPlus tool (24), integrated within SeqSphere+, to identify antimicrobial resistance genes. Multilocus sequence types (STs) for all *Citrobacter* spp. were assigned using the *Citrobacter freundii* MLST scheme (https://pubmlst.org/cfreundii, [25]) as implemented in SeqSphere+. In addition, the species designations were cross-checked with the LPSN database (https://lpsn.dsmz.de/) and were found to explicitly correspond to the recommended designations for medical use.

A comprehensive overview of all isolates and sequences is provided in Table S1. Below is a brief description of the data sets used:

#### Data set 1

GTDB-derived sequences of *Citrobacter* species. These data were used to construct two cgMLST schemes: (i) a *C. freundii*-specific scheme and (ii) a combined scheme covering *C. freundii*, *C. portucalensis*, *C. braakii*, and *C. europaeus*. Species assignments were based exclusively on GTDB classification, comprising 854 *C*. *freundii*, 191 *C*. *portucalensis*, 124 *C*. *braakii*, and 17 *C*. *europaeus* genomes. Two additional *C. freundii* sequences (NCTC9750 and MSB1_1H) were added from NCBI. The *C. portucalensis* data set was expanded with eight sequences generated in this study (see Data sets 4 and 5) and five from the ENA project PRJEB58690 (see Data set 2). Furthermore, 12 *C*. *braakii* and six *C*. *europaeus* sequences were newly assembled and included in the development of the combined scheme.

#### Data set 2

Outbreak-related *C. freundii* and *C. portucalensis* sequences from Finland, retrieved as FASTA files from ENA (PRJEB58690), as described by Heljanko et al. (7).

#### Data set 3

Outbreak-related sequences from Belgium, downloaded as FASTA files from ENA (PRJNA881267), as described by Hamerlinck et al. (6).

#### Data set 4

A total of 24 *Citrobacter* spp. isolates collected and sequenced at UMG (Jan–Aug 2024). These included 20 *C*. *freundii*, and one isolate each of *C. amalonaticus*, *C. europaeus*, *C. braakii*, and *C. portucalensis*. Ten isolates originated from patient samples, and 14 from shower drains in hospital wards. *C. freundii* isolates were analyzed using both cgMLST schemes; the remaining species contributed to development and testing of the combined scheme.

#### Data set 5

A total of 152 *Citrobacter* spp. isolates from the UHoR strain collection (2020–2024), cryopreserved due to multidrug resistance or origin from blood cultures. This set included *C. freundii* (*n* = 96), *C. braakii* (*n* = 13), *C. portucalensis* (*n* = 9), *C. europaeus* (*n* = 5), *C. youngae* (*n* = 12), *C. koseri* (*n* = 10), *C. sedlakii* (*n* = 2), *C. amalonaticus* (*n* = 2), “*C. meridianamericanus”* (*n* = 1), and *C. farmeri* (*n* = 1). Nine isolates were environmental (drains), and 142 were patient-derived. Species identification was performed using MALDI-TOF (Bruker), the Mash Screen tool (26) (SeqSphere+), the AQUAMIS pipeline (20) (using Kraken2 [27]), and TYGS (28); final confirmation used GTDB-Tk. Consensus sequences were generated as described above.

#### Data set 6

*C. portucalensis* sequences from edible snails in Nigeria, downloaded as FASTA files from ENA (PRJNA855947) and described in reference 29.

#### Data set 7

A total of 12 *C*. *europaeus* sequences retrieved from ENA (PRJNA1088829). Ten of these were also included in Data set 1. The phylogenetic analysis of these isolates was described by Ma et al. (30).

### Identification of the *C. freundii* core genome and development of the cgMLST scheme

An *ad hoc* core genome scheme was first created using SeqSphere+ with default settings based on the *C. freundii* type strain ATCC 8090 (GenBank accession CP049015.1, genome size: 4.96 Mb) (31). The selection of valid targets for the *ad hoc* scheme is based on the following criteria. Accessory targets did not meet the following criteria: they occur more than once in at least one reference genome or they overlap within the reference genome. These targets may still be useful for increasing discriminatory power if typing based solely on the MLST+ targets is insufficient. Genes from the reference genome are discarded if (i) genes are shorter than 50 bases; (ii) genes contain no start codon at the beginning of the gene; (iii) genes contain no stop codon; (iv) more than one stop codon or if the stop codon is not at the end of the gene; (v) genes having fragments that occur in multiple copies in a genome (with identity ≥90% and more than 100 bases overlap). Additional criteria can be found on the Ridom SeqSphere+ website (https://www.ridom.de/seqsphere/ug/v40/Core_Genome_MLST_Target_Definer.html). A comprehensive list of all cgMLST targets, including locus tags, gene names, and product descriptions, is provided in Table S5. The genomes from Data set 1 were then screened against this preliminary scheme. Genomes with a total size <4.9 Mb were excluded from downstream analyses.

For the remaining 825 genomes, metadata, such as geographic origin and MLST sequence type (25), were analyzed. Targets identified by SeqSphere+ that were missing in ≥5% of the genomes (marked as “not found” or “failed” in SeqSphere+) were excluded. The remaining loci were retained as core genome targets and included in the final cgMLST scheme. All other loci were assigned to the accessory genome scheme. Target gene coverage was calculated by SeqSphere+ as the total number of base pairs included in all cgMLST targets divided by the total length of the seed genome.

An overview of the scheme development process is illustrated in Fig. S1.

### Development of a combined cgMLST scheme for *C. freundii, C. portucalensis, C. braakii,* and *C. europaeus*

The finalized *C. freundii* cgMLST scheme served as the basis for a combined scheme targeting four species of the *C. freundii* complex. All non-*freundii* genomes from Data set 1 were analyzed using the *C. freundii* scheme to assess the distribution of each target locus across species. To ensure robust interspecies comparability, only those targets present in ≥95% of genomes from each species (*C. freundii*, *C. portucalensis*, *C. braakii*, and *C. europaeus*) were retained for the combined core genome scheme. Remaining loci were included in a corresponding accessory genome scheme. A comprehensive list of all cgMLST targets, including locus tags, gene names, and product descriptions, is provided in Table S5. The calculation of target gene coverage followed the method previously outlined.

The full development workflow is summarized in Fig. S1.

### Software used in this study

Genome assembly, cgMLST, resistance profiling, and tree construction were performed in SeqSphere+. Visualizations were created using SeqSphere+ (MSTs, NJ trees), GraphPad Prism 9, iTOL (https://itol.embl.de), jvenn (32), and Excel 2019 (Microsoft, USA). ChatGPT supported linguistic refinement.

## RESULTS

### Development of a highly specific *C. freundii* cgMLST scheme

**Fig 1:**
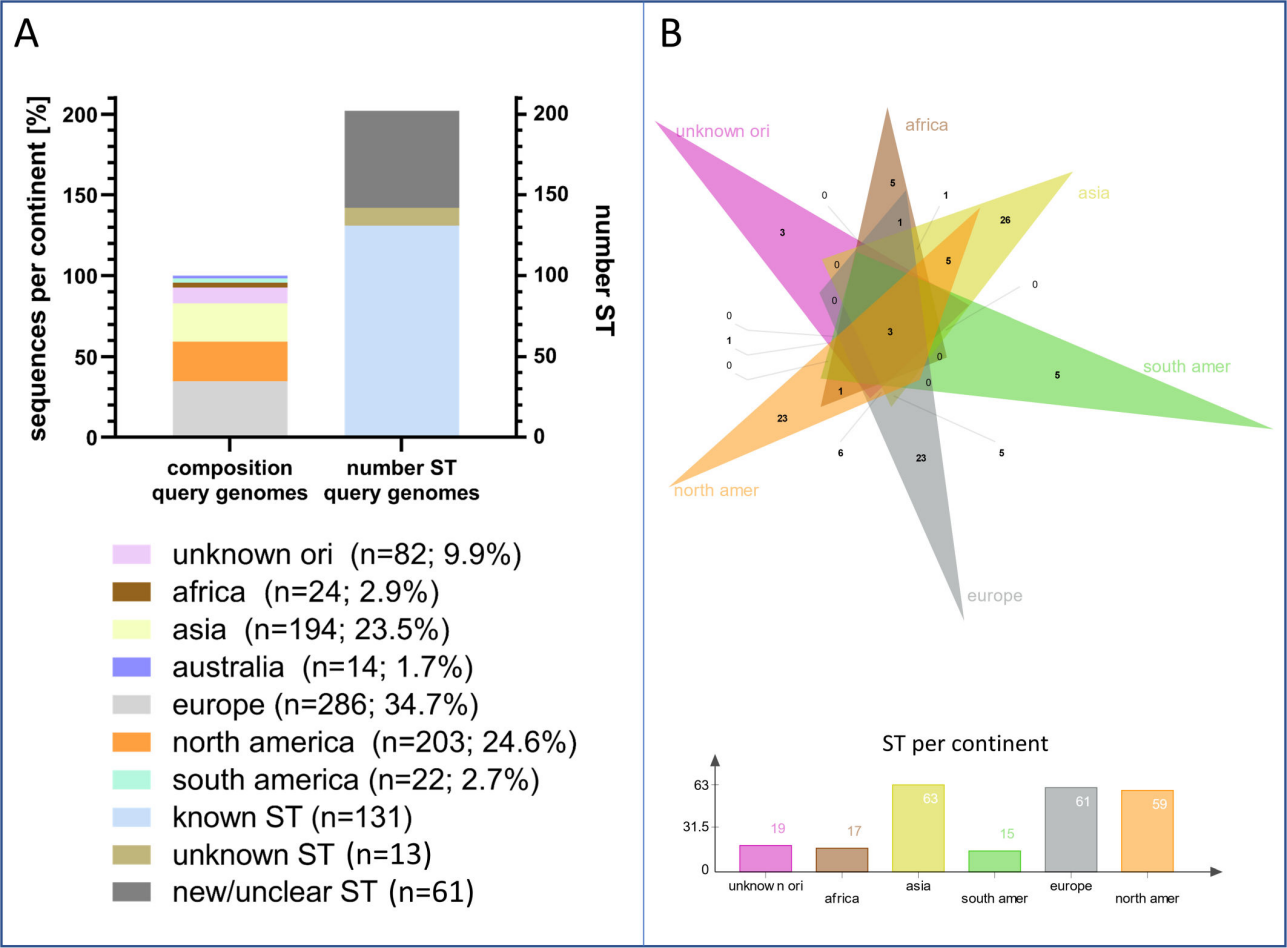
Origin and distribution of *C. freundii* isolates of the GTDB database used to develop the cgMLST scheme. (**A**) The diagram displays the spatial distribution of all isolates based on sequencing metadata obtained from GTDB (left). Additionally, the number of sequence types (STs) identified through MLST analysis using Seqsphere+ (Ridom, Germany) is shown. (**B**) The Venn diagram illustrates the overlap in ST occurrence across continents. The top figure represents the Venn diagram, and the lower chart shows the number of individual ST types per continent. New/unclear STs and unknown STs were each analyzed as a single ST type. The figure was created by GraphPad Prism 9 (**A**) and jvenn (**B**).

**Fig 2:**
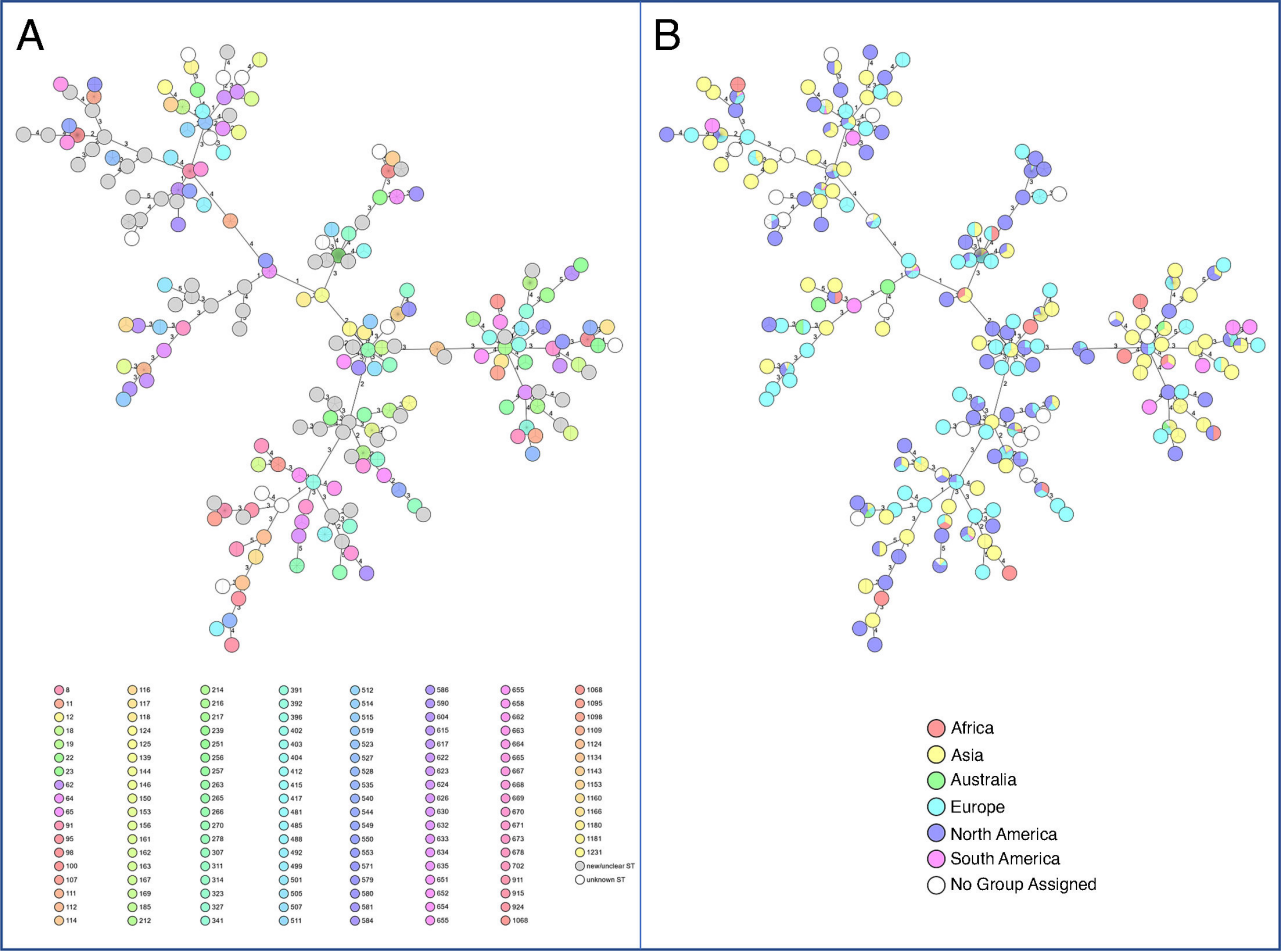
MST of *C. freundii* query genomes based on MLST analysis. (**A**) The MST shows the ST distribution of 825 *C*. *freundii* query genomes. New/unclear STs and unknown STs were each analyzed as a single ST type. (**B**) The same MST displays the distribution of samples according to their origin. To include new/unclear STs and unknown STs in the MST, the “missing values are an own category” function was applied. “No group assigned” indicates missing metadata for these samples. The MST was generated using SeqSphere+ (Ridom, Germany).

To ensure global representativeness, we selected 825 *C*. *freundii* genomes from the curated GTDB database and analyzed them using the *Citrobacter* MLST scheme to assess sequence type (ST) and geographic diversity. These genomes (Data set 1) originated mainly from Europe (*n*  =  286), North America (*n*  =  203), and Asia (*n*  =  194), with fewer from Africa, South America, Australia, and unknown locations (Fig. 1A and B). A total of 133 STs were identified, including 131 known, 13 new/unclear (no matching ST in database found), and 61 novel or ambiguous types (ST assignment impossible: bad sequence quality, no sequence available, or no homolog(s) in database found). While 85 STs appeared to be continent-specific, at least one clearly defined ST (ST22), along with several newly detected or unclassified sequence types, showed indications of global distribution. Minimum spanning trees illustrated broad geographic spread and substantial ST diversity (Fig. 2A and B).

An *ad hoc* cgMLST scheme was generated using the reference strain *C. freundii* ATCC 8090 using SeqSphere+, yielding 4,248 loci (Fig. S1). Of these, 278 were designated accessory and 114 excluded. When applied to the 825 query genomes, 3,250 loci present in ≥95% of isolates were retained as the final cgMLST targets; the accessory scheme included 1,276 loci.

This cgMLST scheme covers ~63% of the ATCC 8090 genome (target gene coverage), a high proportion compared with other species (Table S2), indicating strong core genome stability.

### Validation and evaluation of the *C. freundii* **c**gMLST scheme

**Fig 3:**
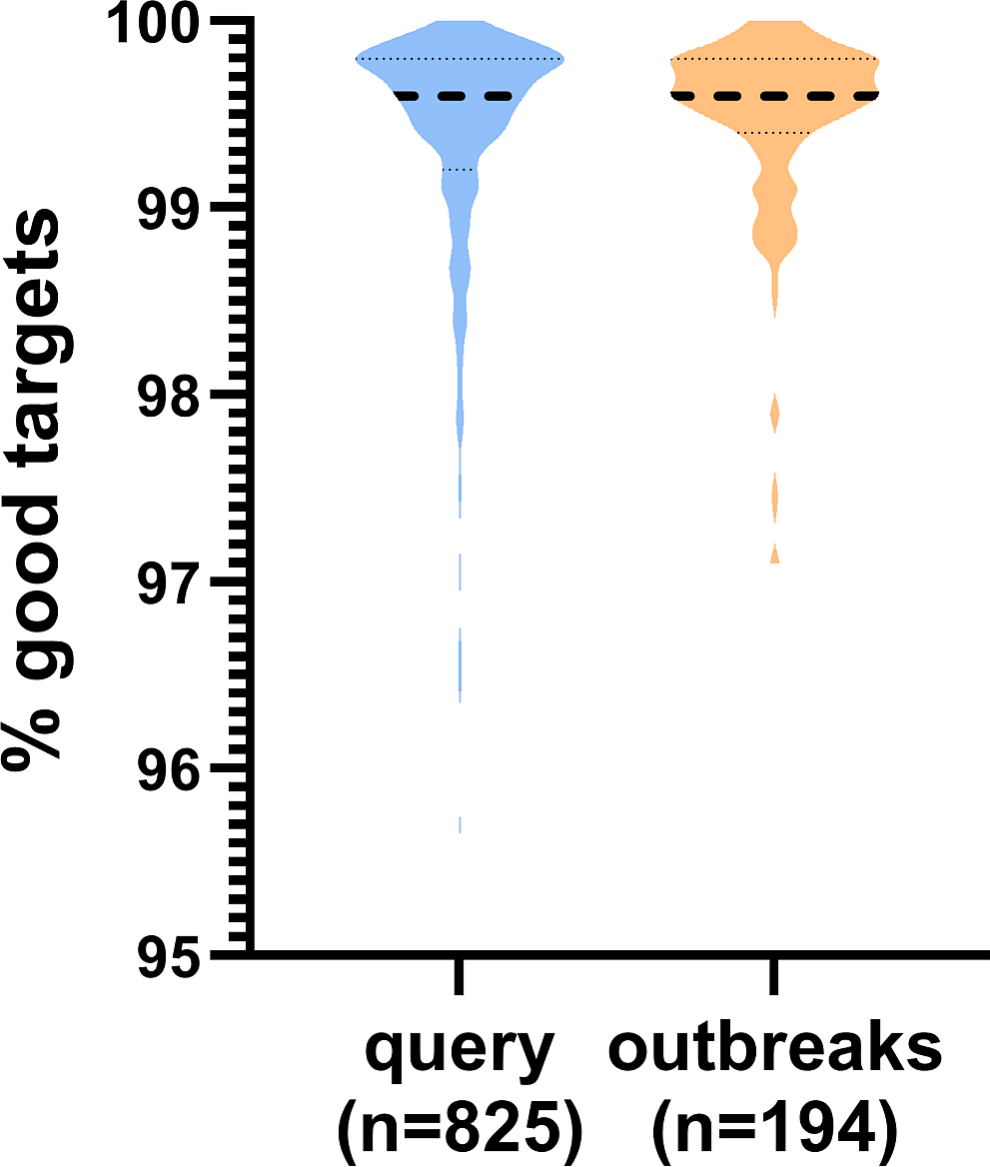
Relative proportion of targets found in cgMLST analyses of query genomes and outbreak genomes. The violin plot illustrates the relative abundance of *C. freundii* cgMLST targets identified in cgMLST analyses (SeqSphere+, Ridom) across query genomes ("query", *n* = 825) and outbreak genomes ("outbreaks," *n* = 194). Dashed line: median, upper dotted lines: Q3 quartiles, lower dotted lines: Q1 quartiles.

To validate the cgMLST scheme, we compared 825 query genomes with 194 additional genomes from previously reported outbreaks (Finland, Belgium) and in-house data from UHoR and UMG (Table S1, data sets 2–5). Median target coverage was 99.6% across all data sets (Fig. 3), indicating robust performance.

In the Finnish outbreak data set (7), seven misclassified genomes (*C. portucalensis* and “*C. meridianamericanus”*) were excluded. These had significantly fewer detected targets (mean: 92.6% and 79.9%, respectively), confirming species-specific performance. For correctly classified *C. freundii* isolates, MST and NJ tree analysis revealed two clusters, ST18 (*n* = 27, Cluster 1) and ST8 (*n* = 19, Cluster 2), in agreement with previous findings. Cluster 1 showed minimal allelic differences (≤10 alleles) (Table S3), supporting likely transmission and validating the 10-allele cluster threshold. Applying the 10-allele threshold to Cluster 2 revealed two distinct subclusters (Fig. S2A).

In the Belgian outbreak data set (6), 21 of 53 isolates identified as *C. portucalensis* were excluded. The remaining 32 *C*. *freundii* genomes revealed one outbreak cluster (Cluster A, ST22) separated from unrelated isolates by >2,600 allele differences (Fig. S2B). Previously reported cluster types D and E were also confirmed. A cgMLST-based NJ tree of all 53 Belgian isolates reproduced the published cluster structure (Fig. S2C), demonstrating the scheme’s high discriminatory power and validity.

### Evaluation of the C*. freundii* cgMLST scheme using clinical and environmental isolates from UMG and UHoR

The validated cgMLST scheme was applied to investigate potential *C. freundii* transmission at the UMG and UHoR. At UMG, 20 isolates were collected between January and August 2024 as part of a screening program for MDR Gram-negative bacteria. Samples originated from patients (*n*  =  6) and shower drains in patient rooms (*n*  =  14).

**Fig 4:**
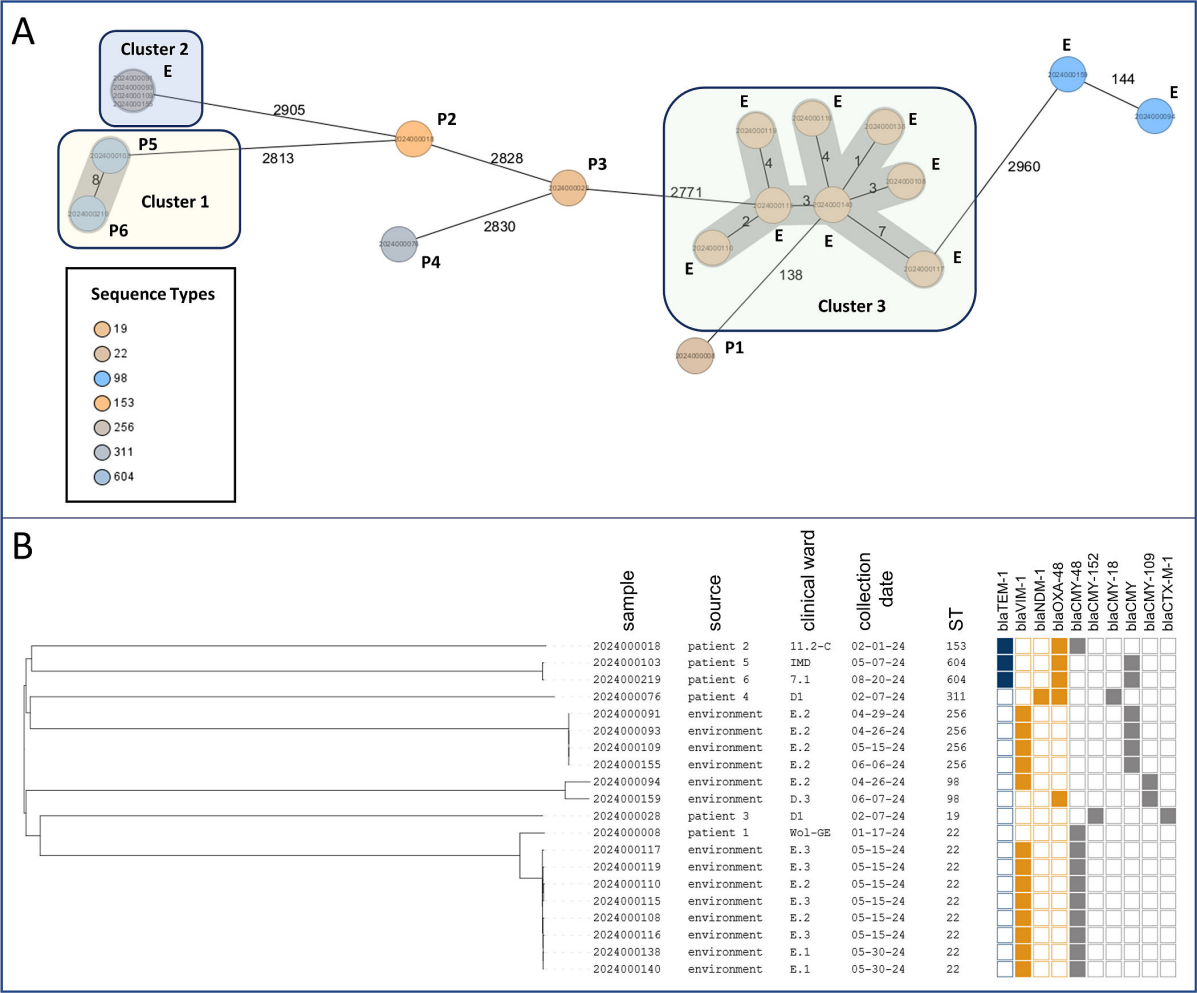
MST and NJ trees from cgMLST analysis of 20 *C*. *freundii* isolates from UMG, Germany. The isolates were obtained during a survey on multidrug-resistant Gram-negative bacteria at the UMG (Germany), comprising six patient-derived (P1–P6) and 14 environmental isolates (E, hospital sinks). Genomes were sequenced and analyzed using the *C. freundii* cgMLST scheme (3,250 targets) in SeqSphere+ (Ridom). (**A**) The MST shows phylogenetic clustering, with three potential outbreak-associated clusters highlighted in transparent red, blue, and green. STs are color-coded; numbers between nodes indicate allelic distances. (**B**) The NJ tree visualizes genetic relationships and sample metadata (isolation source, ward, date, ST). Resistance markers include TEM-1 (blue), carbapenemases VIM-1, NDM-1, OXA-48 (orange), and AmpC/ESBLs such as CMY and CTX-M-1 (gray). Figures were generated using SeqSphere+ (**A**) and iTOL (**B**).

Seven distinct STs were identified, with ST22 (*n*  =  9) and ST256 (*n*  =  4) being the most prevalent (Fig. 4A). cgMLST analysis using the 10-allele threshold revealed three clusters:

Cluster 1 (ST604): two patient isolates (P5, P6) with eight allele differences; both from Ward 7.1, though sampled 3 months apart, suggesting environmental transmission.

Cluster 2 (ST256): four sink isolates obtained from the same ward (2024000091/93/109/155) with no allelic differences.

Cluster 3 (ST22): eight sink isolates from closed clinical wards with 1–7 allelic differences (20240000108/110/115/116/117/119/138/140).

Patient isolate P1 also belonged to ST22 but was excluded from Cluster 3 due to a high allelic distance (138). This genetic divergence aligned with its distinct resistance profile: while Cluster 3 isolates harbored *bla*VIM-1 and *bla*CMY-48, P1 lacked *bla*VIM*-*1 (Fig. 4B).

All other isolates showed ≥2,828 allelic differences and divergent beta-lactam resistance profiles, indicating sporadic occurrence. The two ST98 isolates (2024000094 and 2024000159) were genetically closer (144 alleles apart) but differed in carbapenem resistance.

At UHoR, 263 *Citrobacter* spp. isolates were collected from human samples. A subset of 152 isolates was sequenced, including all MDR strains and a random selection from isolates detected in blood cultures. For select patients (*n* = 24 of 111 patients), follow-up isolates and, in some cases (*n* = 8), multiple colonies per sample were sequenced. Two environmental isolates from toilets were also included, with several colonies sequenced per sample (Table S1, data set 5). Of the 152 sequences (Table S1, Data set 5), 96 were identified as *C. freundii*.

**Fig 5:**
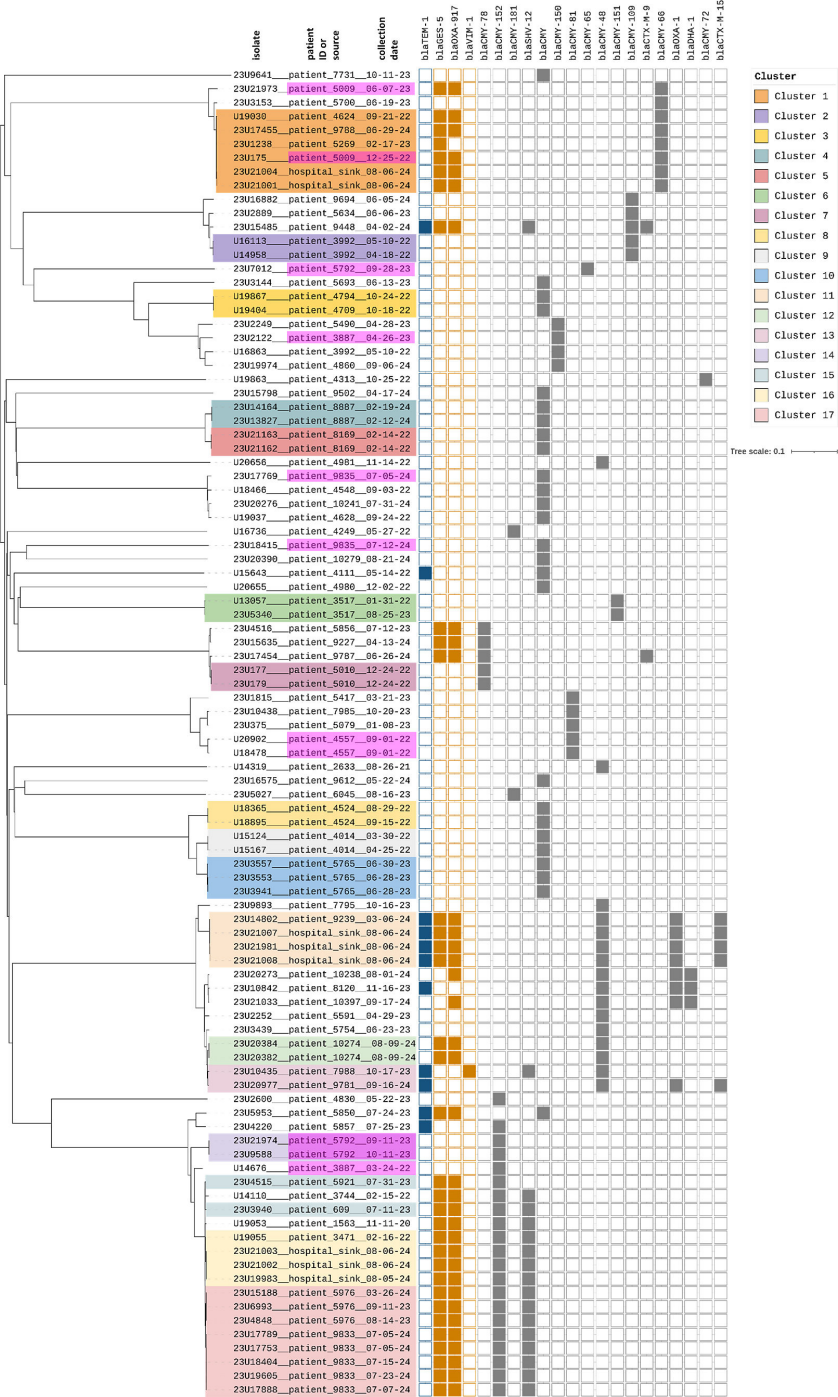
NJ trees derived from cgMLST analyses of 96 *C*. *freundii* isolates from the UHoR, Germany. Clustering of 96 *C*. *freundii* isolates based on cgMLST analysis using a 10-allele threshold. Identified clusters (*n* = 17) are color-coded and annotated with resistance gene profiles (blue: beta-lactam resistance genes, orange: carbapenem resistance genes, gray: cephalosporin resistance genes). Multiple isolates from the same patient (P3887, P4557, P5009, P5792, or P9835) with an allele distance greater than 10 are highlighted in magenta-fuchsia. Consistent resistance profiles within clusters support the chosen threshold. Figure was created by Seqsphere+ (Ridom) (A), B was done with iTOL.

Using the 10-allele cgMLST threshold, 17 clusters were identified (Fig. 5). Cluster validity was supported by concordant resistance gene profiles within clusters, with one exception in Cluster 1.

In total, 16 patients had more than one *C. freundii* isolate sequenced. In five cases, these did not fall into the same cluster (Table S1, data set 5):

Patient 3887 (rectal swab, 13 months apart, two isolates with different STs [ST62, ST19])

Patient 5792 (rectal swab, 19 days apart, three isolates with two different STs [ST928, ST19])

Patient 9835 (rectal swab, 8 days apart, two isolates with different STs [ST540, ST697])

Patient 5009 (rectal swab and abscess, 6 months apart, same STs, but 104 allele difference)

Patient 4557 (two colonies from one swab, same STs, but 18 allele difference)

All other multi-isolate patients showed genetically related strains within clusters (Clusters 2, 4–10, 12, 14, 17), confirming the suitability of the 10-allele threshold.

Two isolates in Cluster 3 (U19867 and U19404) were epidemiologically linked (same ward and timeframe), indicating likely direct transmission. For Cluster 11, patient records indicate a plausible contact between the patient and the sampled toilet. Clusters 1, 13, 15, 16, and 17 cannot be explained by epidemiological links. Notably, toilet-derived isolates clustered with patient isolates, but no direct patient usage near the sampling time could be confirmed. This suggests several possible explanations: undetected carriers, localized environmental reservoirs (e.g., in the wastewater system), or external contamination sources. Follow-up environmental investigations are ongoing.

Overall, transmission of *Citrobacter* spp. at UHoR appears to be rare, but cgMLST enabled the identification of potential clusters and environmental links.

### Development, validation, and application of a combined *Citrobacter* cgMLST scheme

Routine identification methods, such as MALDI-TOF, often misclassify *Citrobacter* species (33, 34), as confirmed in our isolates (Table S1, Data sets 4 and 5) and in published *C. freundii* outbreaks (Table S1, Data sets 2 and 3). As mentioned before, all study genomes (Table S1, Data sets 2-5) were reclassified using GTDB-Tk, revealing frequent misidentifications. Many isolates previously labeled *C. freundii* were reassigned to *C. portucalensis* (*n* = 35), *C. europaeus* (*n* = 5), *C. braakii* (*n* = 2), or others (*n* = 15). To address this, we developed a combined cgMLST scheme targeting four species: *C. freundii*, *C. portucalensis*, *C. europaeus*, and *C. braakii*.

**Fig 6:**
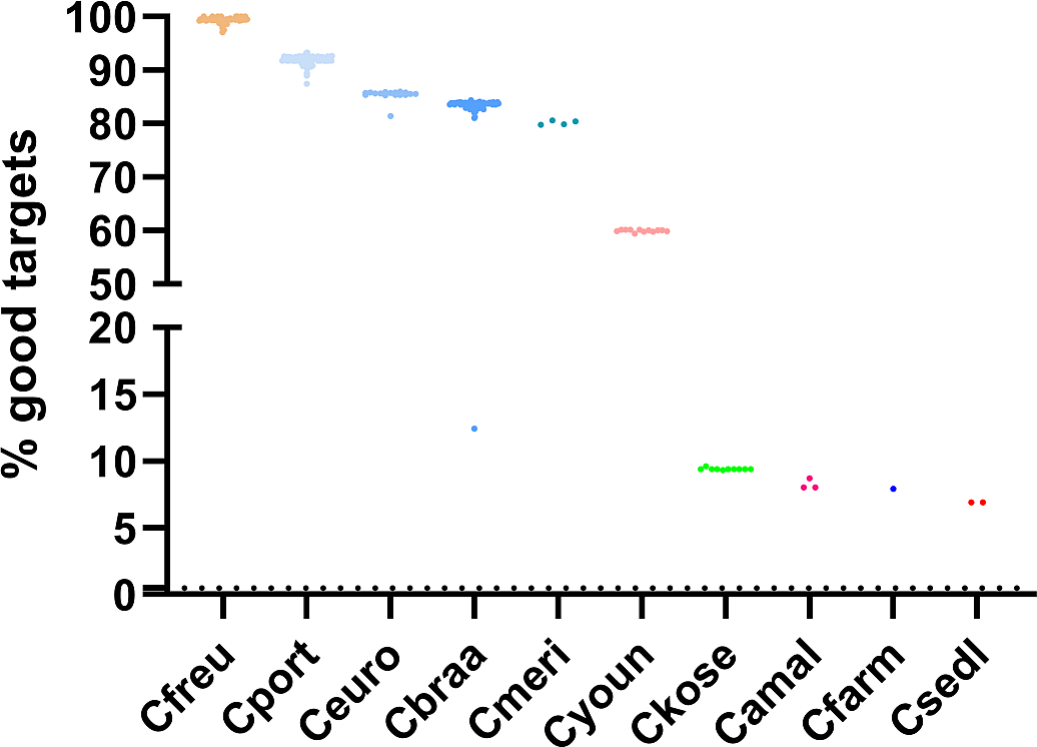
Relative proportion of specific *C. freundii* targets identified in 10 *Citrobacter* species. The violin plot depicts the relative abundance of the 3,250 *C*. *freundii* cgMLST targets detected in cgMLST analyses (SeqSphere+, Ridom) across 10 *Citrobacter* species: *C. freundii* (Cfreu, *n* = 194), *C. portucalensis* (Cport, *n* = 204), *C. europaeus* (Ceuro, *n* = 23), and *C. braakii* (Cbraa, *n* = 136), *C. meridianamericanus* (Cmeri, *n* = 4), *C. youngae* (Cyoun, *n* = 12), *C. koseri* (Ckose, *n* = 10), *C. amalonaticus* (Camal, *n* = 3), *C. farmeri* (Cfarm, *n* = 1), *C. sedlakii* (Csedl, *n* = 2).

To assess cross-species compatibility, we applied the 3,250-target *C. freundii* cgMLST scheme to non-*freundii* isolates. High median target coverage was found in *C. portucalensis* (92.1%), *C. europaeus* (85.6%), *C. braakii* (83.6%), and “*C. meridianamericanus”* (80.2%) (Fig. 6). All other species had significantly lower target gene coverage (<60%) and were excluded. “*C. meridianamericanus”* was also excluded due to low sample number (*n*  =  4).

First, we confirmed MLST-based diversity within *C. portucalensis*, *C. europaeus*, and *C. braakii* query genomes (Fig. S3), and identified 2,307 targets shared by ≥95% of genomes from all four species. These defined the combined core genome; 2,219 loci were assigned to the accessory genome (following workflow Fig. S1).

**Fig 7:**
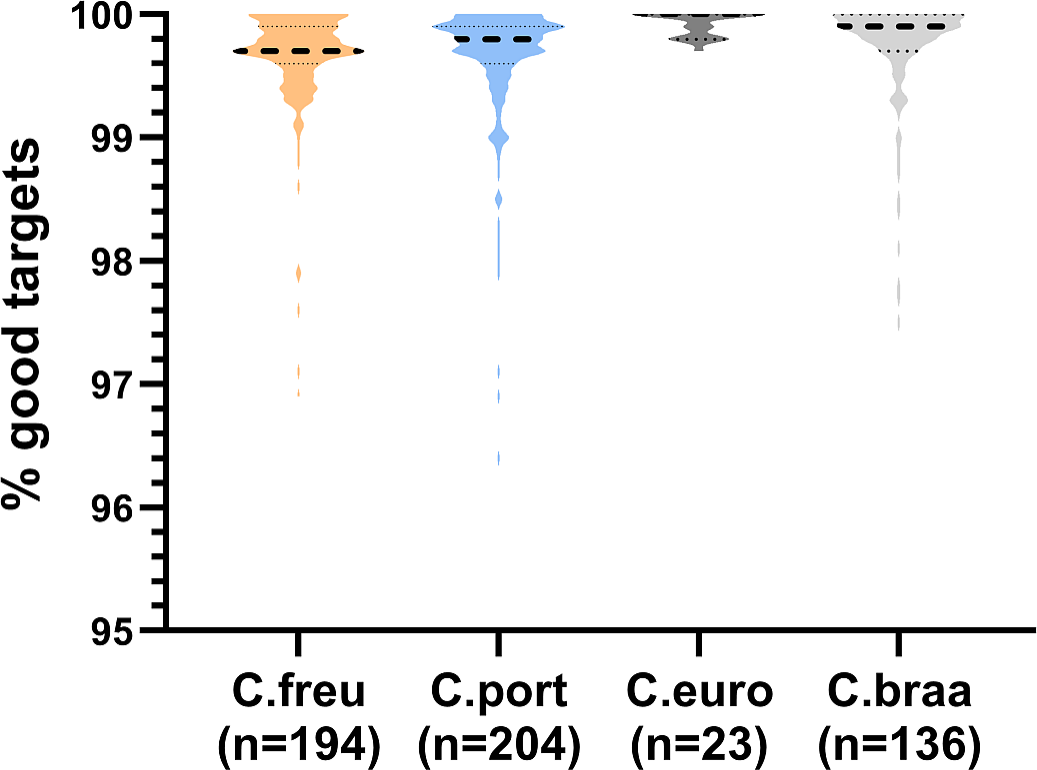
Relative proportion of targets of the combined *Citrobacter* cgMLST scheme in four different *Citrobacter* species. The violin plot depicts the relative abundance of combined-*Citrobacter* cgMLST targets detected in cgMLST analyses (SeqSphere+, Ridom) across four *Citrobacter* species: *C. freundii* (C.freu, *n* = 194); *C. portucalensis* (C.port, *n* = 204), *C. europaeus* (C.euro, *n* = 23), and *C. braakii* (C.braa, *n* = 136). Dashed line: median, dotted lines: Q1 and Q3 quartiles.

All 557 genomes, comprising publicly available sequences from GTDB and ENA as well as newly sequenced clinical and environmental isolates from data sets 1 to 7 (excluding the 825 *C*. *freundii* genomes previously used for development of the *C. freundii*-specific scheme), were reanalyzed using the new combined cgMLST scheme (*C. freundii n*  =  194; *C. portucalensis n*  =  204; *C. europaeus n*  =  23; *C. braakii n*  =  136). The median target coverage was ≥99.7% across all species, indicating excellent applicability (Fig. 7).

Validation included published outbreaks involving *C. freundii* and *C. portucalensis* (6, 7), *C. europaeus* from a Chinese hospital (30) (data set 7), and *C. portucalensis* from edible snails in Nigeria (29) (Table S1, data set 6). No published *C. braakii* outbreak was available; instead, we tested a suspected outbreak at UHoR. Based on the analysis of the *C. freundii* outbreak in Finland, we set the cluster threshold at ≤8 allelic differences (Table S4). The combined scheme successfully reproduced all published outbreak clusters (Fig. S4), including the genetically related *C. portucalensis* snail isolates from Nigeria (two clusters), which clustered closely and supported environmental dissemination potential.

**Fig 8:**
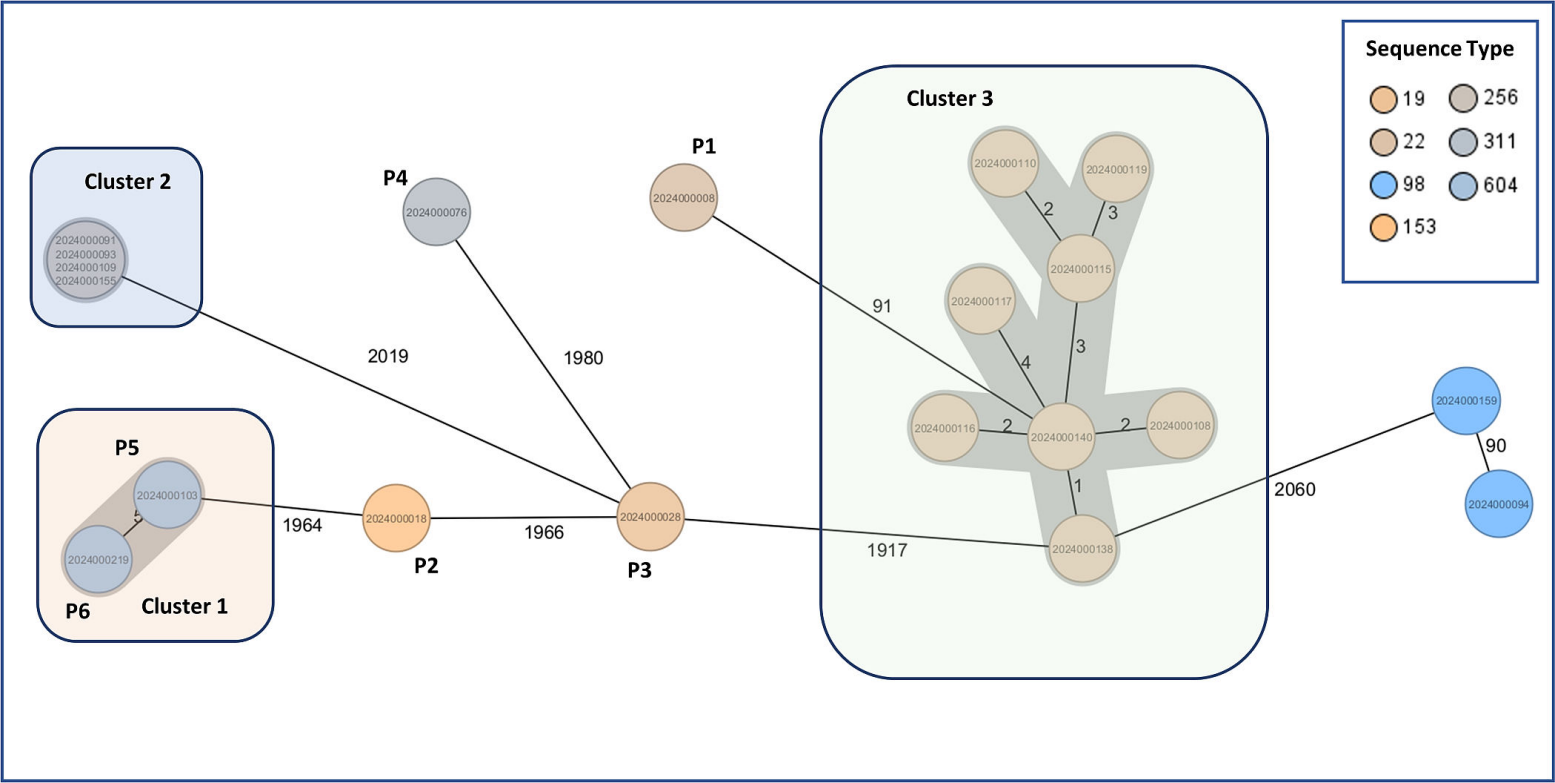
MST from cgMLST analysis of *C. freundii* isolates associated with UMG. Same 20 isolates as described before were used, including patient samples (*n* = 6; P1–P6) and environmental samples from hospital sinks (*n* = 14). cgMLST analysis was performed using SeqSphere+ (Ridom) and the combined *Citrobacter* cgMLST scheme (2,307 targets). Clusters 1 to 3 and their closely related isolates, potentially outbreak-associated, are shown in transparent red, blue, and green. STs are indicated by different node colors; sample IDs are labeled. Unlabeled nodes represent environmental isolates. Numbers on connecting lines indicate allelic distances. Figure generated with SeqSphere+ (Ridom).

**Fig 9:**
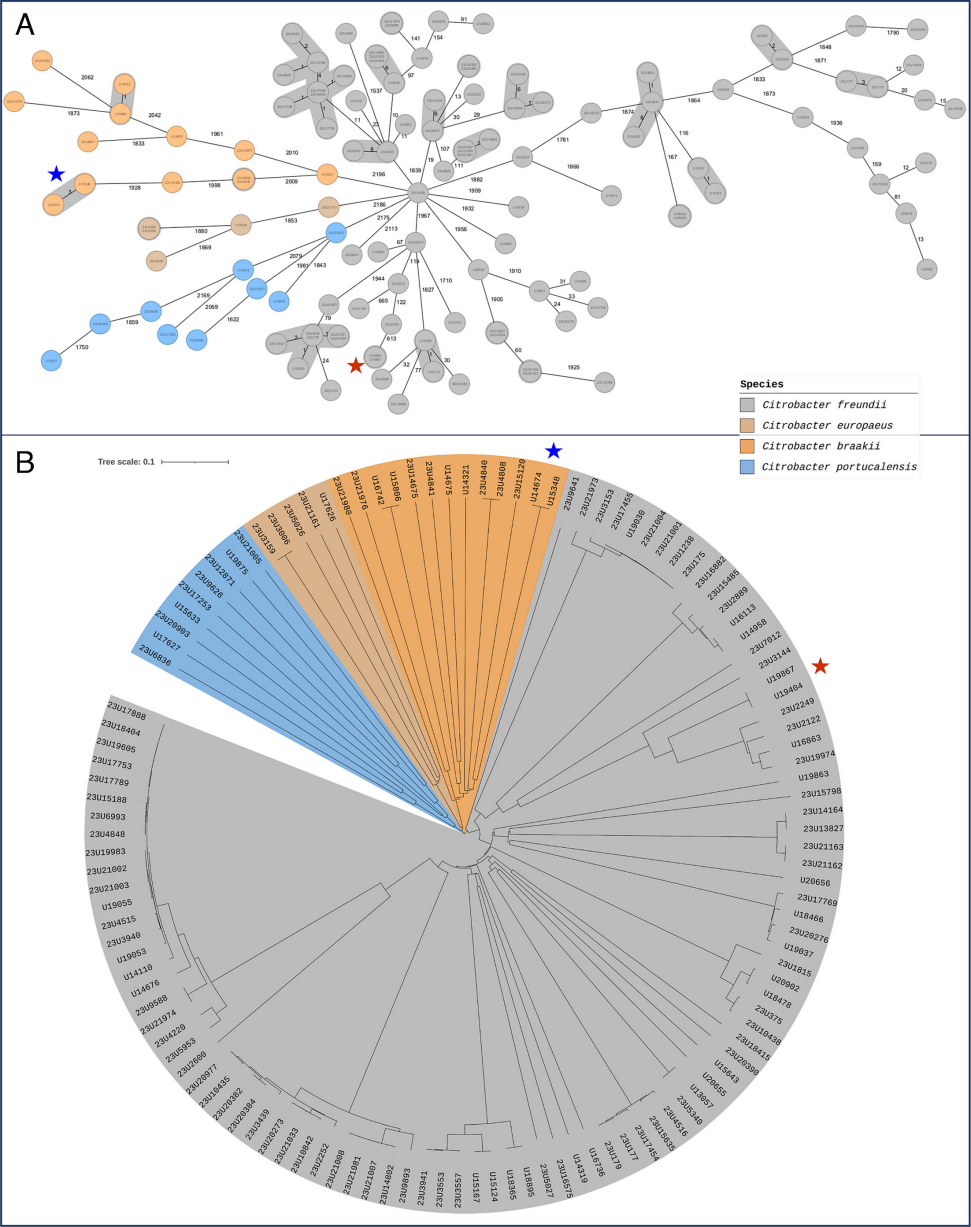
MST and NJ tree of *Citrobacter* spp. isolates from UHoR, based on combined cgMLST scheme. (**A**) MST of isolates from four *Citrobacter* species (*C. freundii, C. braakii, C. europaeus, C. portucalensis*) analyzed using the combined *Citrobacter* cgMLST scheme. A gray background indicates cluster distances ≤8, suggesting phylogenetic relatedness. Species are color-coded as shown in the legend. (**B**): NJ tree of the same analysis. *C. freundii* forms distinct clusters, while non-*freundii* species show high divergence with long branches. Identical *C. europaeus* isolates (0 alleles) originated from the same patient. Three *C. braakii* isolate pairs were closely related (allelic distances 0–1), including one inter-patient pair from the same ward. Possible transmissions are marked with stars: blue for *C. braakii*, brown for *C. freundii*.

We then applied the scheme to isolates from UMG (Fig. 8) and UHoR (Fig. 9). At UMG, the same three *C. freundii* clusters identified by the species-specific scheme were detected with adjusted allele distances due to fewer targets (2,307 vs 3,250). Non-*freundii* species from UMG (*n*  =  4) were too few for cgMLST-based conclusions.

At UHoR, sufficient numbers of *C. freundii* (*n*  =  96), *C. portucalensis* (*n*  =  9), *C. europaeus* (*n*  =  5), and *C. braakii* (*n*  =  13) allowed full analysis. MST and NJ trees showed well-defined *C. freundii* clusters, while other species were more diverse. Among *C. europaeus*, two identical sequences from one patient (23U3006, 23U3159) showed 0 allelic differences. For *C. braakii*, three sequence pairs were detected:

Two intra-patient pairs (0 and 1 allele difference)

One inter-patient pair (U14674/U15348), differing by three alleles, was collected from the same ward at the same time, suggesting a possible transmission event.

These findings confirm that the combined *Citrobacter* cgMLST scheme enables accurate cross-species surveillance, including outbreak detection and environmental tracking, even across geographic regions and species boundaries.

## DISCUSSION

Although *Citrobacter* spp. have historically been considered as relatively low-virulence pathogens, recent years have seen a rise in nosocomial infections and hospital outbreaks, particularly involving *C. freundii* (3, 35, 36). To date, a variety of typing approaches, including PFGE, MLST, *ad hoc* cgMLST, and SNV-based methods, have been used for outbreak investigation (5–7, 37–47). However, genome-wide gene-by-gene typing via cgMLST offers a standardized, scalable, and high-resolution alternative (48–52). Despite this, no validated cgMLST scheme for *Citrobacter* spp. existed prior to this study.

We therefore developed two schemes: one species-specific for *C. freundii* (3,250 targets) and one combined scheme for *C. freundii*, *C. portucalensis*, *C. braakii*, and *C. europaeus* (2,307 targets). The *C. freundii* scheme demonstrated high robustness, with 99.6% of targets detected in 1,019 genomes from globally diverse sources. Its 63% genome coverage (based on the ATCC 8090 reference) outperforms many existing schemes (Table S2). Applying this scheme to previously published outbreaks in Finland and Belgium (6, 7) reproduced clustering results and even increased resolution, particularly in the Finnish ST8 cluster. A maximum intra-cluster distance of 10 alleles was established as a transmission threshold, consistent with previous reports (7). These published outbreaks provided a critical validation benchmark for the newly developed scheme, ensuring that its performance was tested against well-characterized nosocomial outbreaks.

This threshold was applied to outbreak investigations at UMG and UHoR. At UMG, no direct patient-to-patient transmission was confirmed. However, genetically related isolates with identical resistance genes were found in patients and in shower drains, indicating possible environmental reservoirs. A similar pattern emerged at UHoR, where environmental isolates from toilets, sinks, and drains were genetically indistinguishable from patient strains, even after >2 years. These findings suggest long-term persistence in sanitary systems and support environment-to-patient transmission, with human-to-human spread being rare but possible.

Species misclassification is common with conventional methods like MALDI-TOF (33, 34), complicating outbreak investigations. By applying GTDB-Tk, we revealed extensive mislabeling of *Citrobacter* spp. in public data sets. Although no formal nomenclatural definition of the “*C. freundii* complex” exists, this designation is commonly used in the literature and taxonomic resources to describe a phylogenetically coherent group comprising *C. freundii*, *C. portucalensis*, *C. braakii*, and *C. europaeus*. This grouping is supported by genome-based analyses, including ANI and whole-genome phylogenetics (34, 53, 54). The NCBI Taxonomy database includes an entry for the “*Citrobacter freundii* complex” (https://www.ncbi.nlm.nih.gov/Taxonomy/Browser/wwwtax.cgi?id=1344959) but defines it more broadly and encompasses additional species, such as *C. youngae* and *C. sedlakii*. Our analyses explicitly demonstrate that both species exhibit substantially divergent core genomes and therefore could not be included in the combined scheme. In this study, we focused on *C. freundii*, *C. portucalensis*, *C. braakii*, and *C. europaeus* due to their close phylogenetic relationship and clinical relevance, which facilitates the development of a robust cross-species cgMLST scheme. Our combined scheme, though containing fewer targets than the *C. freundii*-specific one, maintained high median target recovery (≥99.7%) across the four species.

The combined scheme successfully resolved transmission clusters in previously published outbreaks, including those involving *C. portucalensis* in humans (6, 7) and in edible snails from Nigeria (29), and *C. europaeus* in a Chinese hospital (30). At UHoR, the scheme enabled detailed analysis of *C. portucalensis*, *C. braakii*, and *C. europaeus* isolates, confirming their environmental persistence and potential for transmission. Only one potential patient-to-patient transmission event was identified, again pointing to environmental reservoirs as dominant sources. The finding of *C. portucalensis* in sinks supports the hypothesis of surface-mediated transmission via water systems, shared facilities, or healthcare workers (3, 6, 7, 47).

Together, these results demonstrate that our cgMLST schemes allow for high-resolution outbreak tracking of *Citrobacter* spp., even when species identification is uncertain. They provide powerful tools for infection control and molecular surveillance in clinical settings.

### Limitations

A limitation of this study is that the UHoR data set included almost exclusively patient isolates, without corresponding environmental sampling. This restricts the ability to identify reservoirs or reconstruct transmission pathways—an important aspect when studying *Citrobacter* spp., which are frequently associated with environmental niches. In addition, the clinical collections from UMG and UHoR reflected different inclusion criteria, as they were assembled for routine surveillance rather than targeted outbreak investigations. To mitigate this limitation, we included published, well-characterized nosocomial outbreak data sets from Finland and Belgium as validation cohorts. The need for integrated patient and environmental sampling also applies to other data sets analyzed with the newly developed cgMLST schemes.

### Conclusions

We developed and validated two cgMLST schemes: a high-resolution, species-specific scheme for *C. freundii* and a combined scheme for four closely related *Citrobacter* species. The *C. freundii* scheme reliably detects outbreak clusters and supports transmission analysis. The combined scheme enables cross-species outbreak investigations, even when species-level classification is uncertain, and is particularly suited for routine infection control. Given the rising clinical relevance and multidrug resistance of *Citrobacter* spp., both schemes provide essential tools for genomic surveillance, source tracking, and future studies on the ecology and evolution of this genus.

## Data Availability

Newly assembled consensus sequences from this study were deposited in the National Center for Biotechnology Information (NCBI; https://www.ncbi.nlm.nih.gov/) under the study accession numbers PRJNA1266399 (UMG) and PRJNA1204071 (UHoR).

## SUPPLEMENTAL MATERIAL

Supplemental figures jcm.00860-25-s0001.pdf Fig. S1 to S4.

Table S1 jcm.00860-25-s0002.xlsx Comprehensive overview of all isolates and sequences.

Table S2 jcm.00860-25-s0003.xlsx Relative genome coverage of the Citrobacter cgMLST schemes compared to some other pathogens.

Table S3 jcm.00860-25-s0004.xlsx Distance matrix of cluster 1 *C. freundii* cgMLST scheme (3,250 targets).

Table S4 jcm.00860-25-s0005.xlsx Distance matrix.

Table S5 jcm.00860-25-s0006.xlsx cgMLST targets of the *C. freundii* cgMLST scheme.

ASM does not own the copyrights to Supplemental Material that may be linked to, or accessed through, an article. The authors have granted ASM a non-exclusive, world-wide license to publish the Supplemental Material files. Please contact the corresponding author directly for reuse.
