# Supplementary material for: Development and validation of a core genome multilocus sequence typing scheme for Citrobacter freundii: application in outbreak investigations and comparative analysis across the Citrobacter genus
Source: J Clin Microbiol. 2025 Sep 19;63(10):e00860-25. doi: 10.1128/jcm.00860-25 (PMC12505962; doi:10.1128/jcm.00860-25)
Supplement: Supplemental figures — Fig. S1 to S4. [file jcm.00860-25-s0001.pdf]

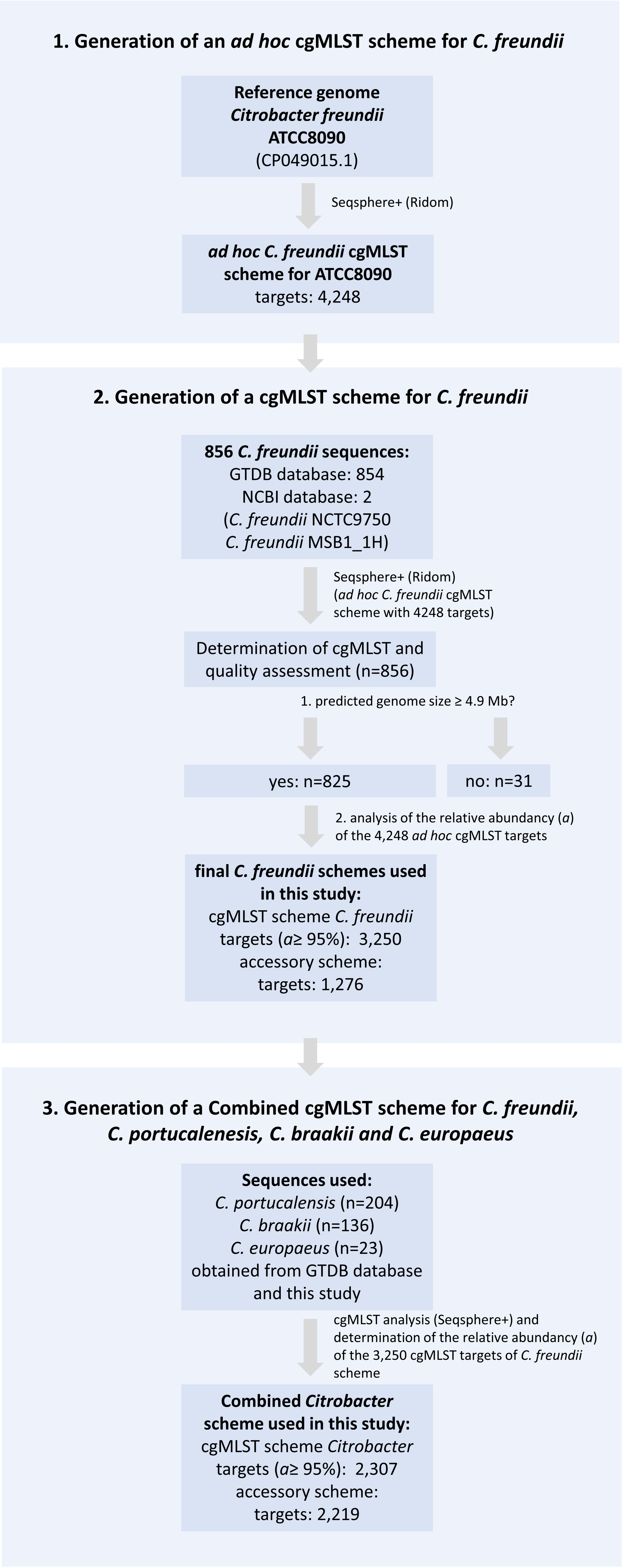

Figure S1. Flowchart for the development of cgMLST schemas for *C. freundii* alone and combined for *C. freundii*, *C. portucalensis*, *C. braakii*, and *C. europaeus.*

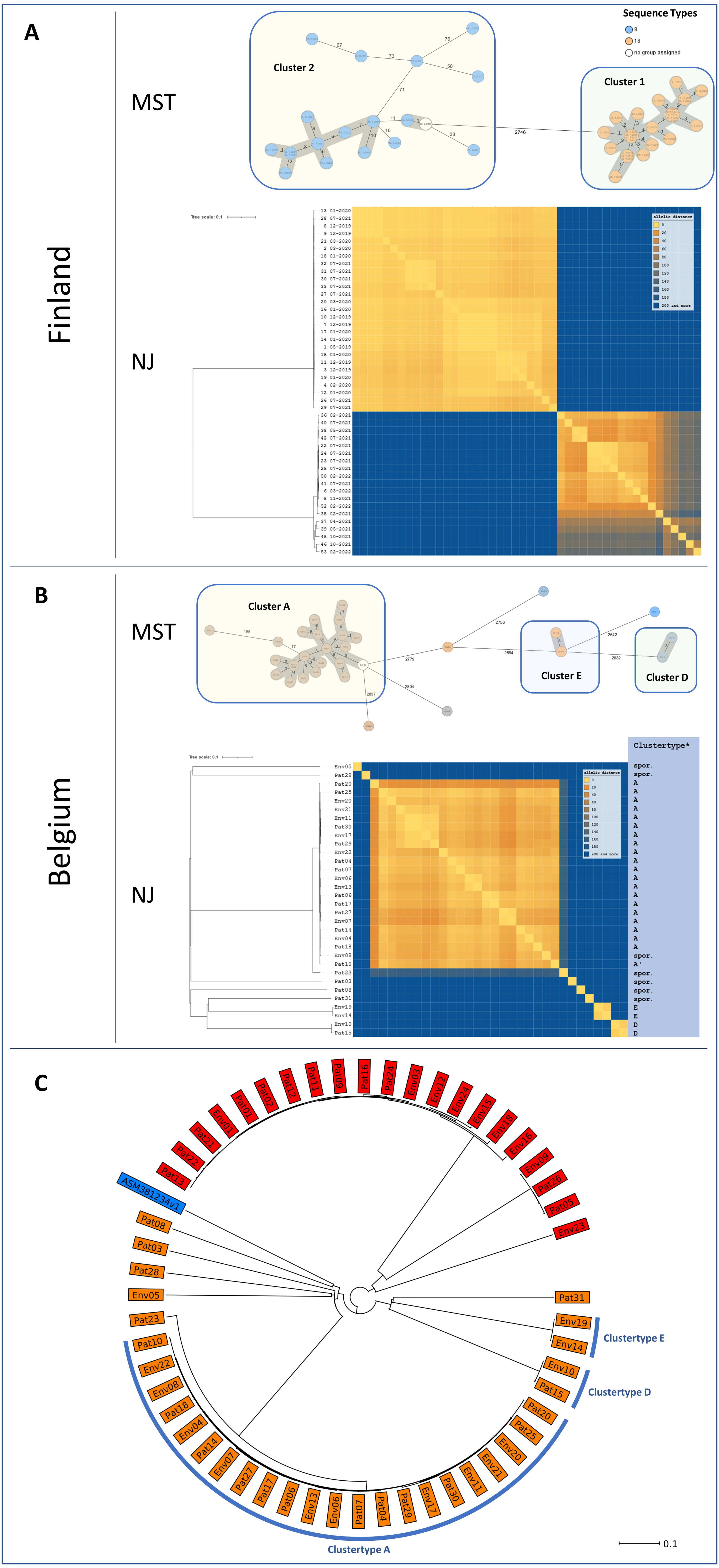

Figure S2. Reanalysis of data from Finland (**A**) and Belgium (**B**, **C**): Top panels show the minimum spanning tree (MST) generated with SeqSphere+ (Ridom), while the bottom panels display the neighbor-joining tree constructed using SeqSphere+ and iTOL, accompanied by the allele difference matrix visualized as a heatmap. Isolates shaded in gray in the MSTs have allele distances ≤10 and are considered potential outbreak strains. **C**: Neighbour joining tree (NJ) of a *Citrobacter* spp. outbreak based on cgMLST analysis with the *C. freundii* cgMLST scheme developed in this study. Analysis was done with Seqsphere+ (Ridom, Germany) using the 53 outbreak and one reference sequences of an *Citrobacter* spp. outbreak described in Belgium. GTDB-Tk tool was used for species verification/identification (orange: *C. freundii* outbreak strains, blue: *C. freundii* reference strain NZ_CP033744.1 (ASM381234v1); red: *C. portucalensis* outbreak strains). References: Finland (A): Heljanko *et al*., 2023; Belgium (B,C): Hamerlinck *et al*., 2023

Heljanko, V., et al., Genomic epidemiology of nosocomial carbapenemase-producing *Citrobacter freundii* in sewerage systems in the Helsinki metropolitan area, Finland. Front Microbiol, 2023.14:1165751
Hamerlinck, H., *et al*., Sanitary installations and wastewater plumbing as reservoir for the long-term circulation and transmission of carbapenemase producing *Citrobacter freundii* clones in a hospital setting. Antimicrob Resist Infect Control, 2023. 12(1): p. 58.

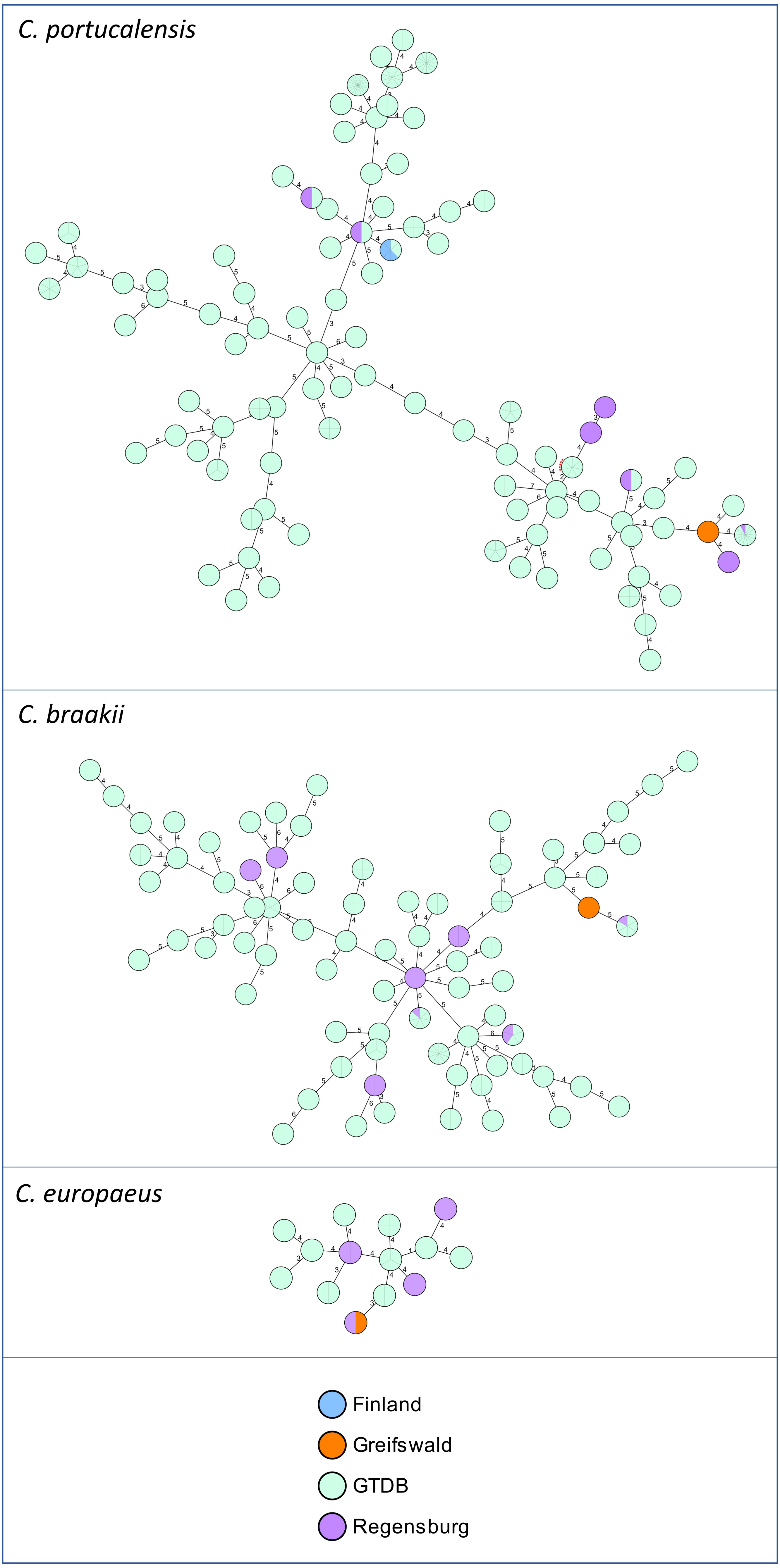

Figure S3. MLST analysis of *C. portucalensis*, *C. braakii*, and *C. europaeus* query genomes. The MSTs illustrate the sequence type (ST) distribution of 204 *C. portucalensis*, 136 *C. braakii*, and 23 *C. europaeus* query genomes, though individual STs are not shown. The MLST analyses were performed using the *Citrobacter* spp. MLST scheme (Bai *et al*., 2012). This approach effectively captures the extensive phylogenetic diversity of the query genomes, which were subsequently used to develop the final combined-*Citrobacter* cgMLST scheme for analyses of *C. freundii*, *C. portucalensis*, *C. braakii* and *C. europaeus*. In addition to query sequences obtained from the GTDB database (mint green), the analysis incorporated sequences from this study (Greifswald (UMG) orange, and Regensburg (UHoR) purple) as well as sequences from a *Citrobacter* outbreak in Finland (Heljanko *et al.*, 2023). The MSTs were generated using SeqSphere+ (Ridom, Germany), with the 'Missing values are an own category' function applied.

Bai, L., *et al*., Isolation and characterization of cytotoxic, aggregative *Citrobacter freundii*. PLoS One, 2012. 7(3): p. e33054.
Heljanko, V., *et al*., Genomic epidemiology of nosocomial carbapenemase-producing *Citrobacter freundii* in sewerage systems in the Helsinki metropolitan area, Finland. Front Microbiol, 2023. 14: p. 1165751.

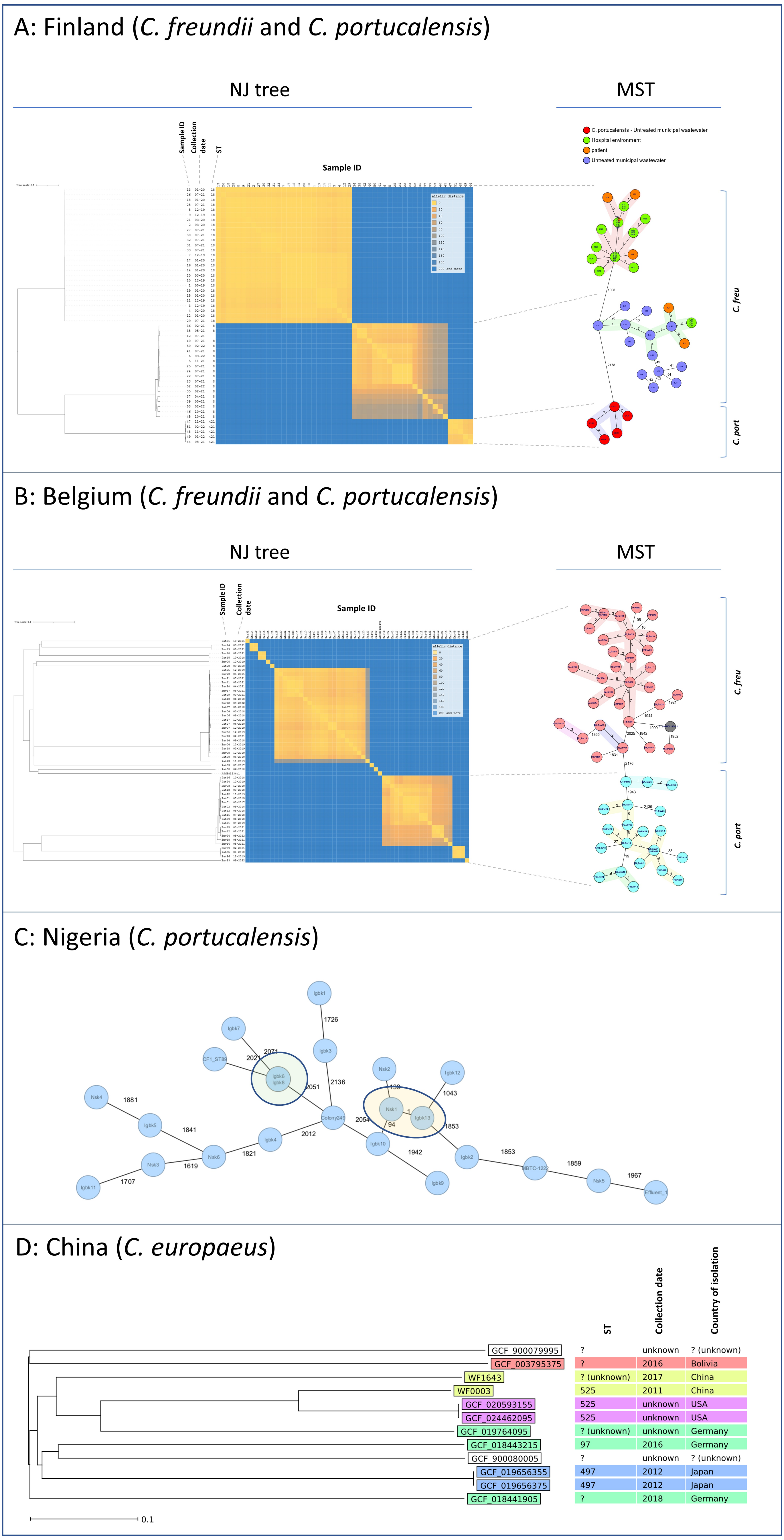

Figure S4. Reanalyzes of *Citrobacter* spp. outbreak studies using the combined-*Citrobacter* cgMLST scheme. The new analyses included the *Citrobacter* spp. outbreak studies and/or environmental screenings of Finland (A) (Heljanko *et al.*, 2023), Belgium (B) (Hamerlinck *et al.*, 2023), Nigeria (C) (Okafor *et al.*, 2024), and China (D) (Ma *et al.*, 2024). Shown are NJ trees and corresponding MSTs (A, B), only MST (C), and only an NJ tree (D). **A and B**: The NJ tree and matrix were created using iTOL, while the MST was generated with SeqSphere+ (Ridom). The colored backgrounds in the MSTs indicate close phylogenetic relationships with allele distances ≤8, and the corresponding allele distances are annotated on the connecting lines. Individual isolates are labeled with their ST and sample ID. **C:** Shown is the cgMLST based-MST calculated for an environmental screening in Nigeria (Africa) of *C. portucalensis* in snails. Two clusters of isolates that are phylogenetically closely related are highlighted. The results confirm the findings of Okafor *et al*. (2024). The MST was created using SeqSphere+ (Ridom). **D:** Displayed is the NJ tree of cgMLST analyses using the same *C. europaeus* sequences analyzed by Ma *et al*. (2024). Highly similar results were obtained. The NJ tree was generated using SeqSphere+ (Ridom).

Heljanko, V., *et al*., Genomic epidemiology of nosocomial carbapenemase-producing *Citrobacter freundii* in sewerage systems in the Helsinki metropolitan area, Finland. Front Microbiol, 2023. 14: p. 1165751.
Hamerlinck, H., *et al*., Sanitary installations and wastewater plumbing as reservoir for the long-term circulation and transmission of carbapenemase producing *Citrobacter freundii* clones in a hospital setting. Antimicrob Resist Infect Control, 2023. 12(1): p. 58.
Okafor, A.C., *et al*., Antibiotic Resistance Hotspot: Comparative Genomics Reveals Multiple Strains of Multidrug-Resistant *Citrobacter portucalensis* in Edible Snails. Int J Mol Sci, 2024. 25(18).
Ma, J., *et al*., Whole-genome sequencing of clinical isolates of *Citrobacter europaeus* in China carrying *bla*(OXA-48) and *bla*(NDM-1). Ann Clin Microbiol Antimicrob, 2024. 23(1): p. 38.
